# Investigating the mechanism by which *SMAD3* induces *PAX6* transcription to promote the development of non-small cell lung cancer

**DOI:** 10.1186/s12931-018-0948-z

**Published:** 2018-12-29

**Authors:** Zhe Qian, Qiankun Zhang, Ying Hu, Tongmei Zhang, Jie Li, Zan Liu, Hua Zheng, Yuan Gao, Wenyun Jia, Aimin Hu, Baolan Li, Jiqing Hao

**Affiliations:** 1 Department of General Medicine, Beijing Chest Hospital, Capital Medical University & Beijing Tuberculosis and Thoracic Tumor Research Institute, No.9 Yard, Beiguan Street, Tongzhou District, Beijing, 101149 China; 2 Department of Medical Oncology, The First Affiliated Hospital of Anhui Medical University, No. 218 Jixi Road, Shushan District, Hefei, 230022 Anhui China

**Keywords:** Non-small cell lung cancer, *SMAD3*, *PAX6*

## Abstract

**Background:**

This study investigated the function of *SMAD3* (SMAD family member 3) in regulating *PAX6* (paired box 6) in non-small cell lung cancer.

**Methods:**

First, qRT-PCR was employed to detect *SMAD3* expression in cancer tissues along with normal tissues and four cell lines, including BEAS-2B, H125, HCC827 and A549 cells. *SMAD3* was knocked down by small interference RNA (siRNA), and then its expression was determined via qRT-PCR and Western blot analysis. The correlation between SMAD3 and PAX6 was determined by double luciferase reporter experiments and chromatin immunoprecipitation (ChIP) assay. Cell viability was evaluated by CCK-8 and colony forming assays, while cell migration and invasion were detected by Transwell analysis.

**Results:**

*SMAD3* and *PAX6* were upregulated in lung cancer tissues and cancer cells. Knocking down *SMAD3* and *PAX6* by transfection with siRNAs specifically suppressed the expression of *SMAD3* and *PAX6* mRNA and protein levels. SMAD3 could promote *PAX6* transcriptional activity by binding to its promoter. Reduced expression of SMAD3 led to the downregulation of PAX6 mRNA and protein levels along with decreased cell migration, invasion, proliferation and viability in A549 and HCC827 cells. *PAX6* overexpression altered the si-SMAD3-induced inhibition of cell migration, invasion, proliferation and viability in A549 and HCC827 cells. Additionally, *PAX6* knockdown alone also repressed the cell migration, invasion, proliferation and viability of the cell lines.

**Conclusions:**

*SMAD3* promotes the progression of non-small cell lung cancer by upregulating *PAX6* expression.

**Electronic supplementary material:**

The online version of this article (10.1186/s12931-018-0948-z) contains supplementary material, which is available to authorized users.

## Background

One of the most common cancers is non-small cell lung cancer (NSCLC), which accounts for 1/4 cancer-related mortalities each year [1]. Tyrosine kinase inhibitors and surgical resection are common treatments for patients with NSCLC, but, in the virtue of drug resistance or compromising cardiopulmonary reserves, the effects of treatment are poor [2, 3]. Furthermore, a novel method for treating lung cancer, stereotactic body radiation therapy, could shorten the treatment course due to high doses of radiation and precise targeting [4]. Although many advances in cancer research have been made, the prognosis of NSCLC is still unsatisfactory, with a lower 5-year survival rate compared with other cancers [5]. Consequently, investigating the pathogenesis of NSCLC might give us a chance to discover impactful and effective treatment methods for NSCLC, and has grown in importance.

Previous investigations have showed that members of the transforming growth factor beta (TGF-β) superfamily and their associated downstream signaling components, SMADs, play a crucial role in several aspects of breast cancer onset and disease progression [6]. The role of Smad3 in many cancers is an emerging area of intense research. According to a former study, *SMAD3* might contribute to increasing the risk of breast cancer by encoding a key protein that interacts with *BRCA2* [7]. Moreover, Li et al. reported that the deregulation of *SMAD3* expression was associated with ventricular septal defects [8]. Meanwhile, some studies have focused on uncovering the correlation between *SMAD3* and lung cancer. For example, Samanta et al. reported that reducing *SMAD3* expression could abrogate TGF-β-mediated growth inhibition, resulting in promoting tumorigenicity [9]. Previous studies have shown that SMAD3 is involved in aggressive tumor behavior in NSCLC and might act as a potential target for the treatment of the cancer [10]. A published paper reported that downregulating TGFBR2 expression promoted the proliferation, migration and invasion of NSCLC cells by reducing the activation and phosphorylation of Smad2 and Smad3 [11]. Thus, the elusive mechanisms involving *SMAD3* in the development and progression of NSCLC deserve more attention.

Paired box (PAX) proteins play a crucial role in normal embryogenesis, which can regulate cell proliferation, self-renewal and apoptosis and even participate in the migration of embryonic precursor cells as well as differentiation programs [12]. There is an emerging hypothesis that PAX proteins might inhibit terminal differentiation and apoptosis in issue-specific stem cells, resulting in maintaining these cells [13]. This effect is likely to be involved in cancer cell development and progression. Moreover, *PAX6,* a paired box family gene, was recently demonstrated to be involved in the development of pancreatic neuroendocrine tumors [14]. Furthermore, in the investigation by Li et al., *PAX6* expression had been proven to be suppressed by microRNA-7 in human colorectal cancer cells, resulting in inhibited cell proliferation and invasion [15]. Similarly, Luo et al. had suggested that miR-7 negatively regulates PAX6 protein levels, which can promote the proliferation and invasion of NSCLC cells via activation of the ERK and MAPK signaling pathways [16]. Kiselev et al. also showed that the transcription factor PAX6 was a novel prognostic factor and putative tumor suppressor in non-small cell lung cancer [17]. Pax6 also interacts with the Smad3 MH1 domain, and Pax6/Smad3 interactions appear to be necessary for TGF-β signaling [18]. Tripathi et al. also indicated the involvement of SPARC in the Smad3-dependent autoregulation of Pax6 to complete the loop and interact with Smad3 [19]. However, deeper investigation and discussion on SMAD3 and PAX6 in NSCLC cells is still needed.

In this study, we investigated the function of *SMAD3* in non-small cell lung cancer using cell proliferation and migration experiments and explored the relationship between *SMAD3* and *PAX6* with double luciferase reporter experiments and chromatin immunoprecipitation assay (ChIP).

## Methods

### Clinical tissue samples

The 20 NSCLC tissue samples and 20 normal tissues examined in the experiments were provided by Beijing Chest Hospital, Capital Medical University & Beijing Tuberculosis and Thoracic Tumor Research Institute. Histopathological types were assigned using WHO pathological staging criteria. The 20 tumor tissues used in our study were adenocarcinomas. Frozen tissue was used in our study. All patients investigated were not treated with preoperative chemotherapy or radiotherapy. The Ethics Committee of Beijing Chest Hospital, Capital Medical University & Beijing Tuberculosis and Thoracic Tumor Research Institute and the patients have approved the experiments in the present study.

### Cell cultures

The normal human lung epithelial cells, BEAS-2B, and cancer cell lines, H125, HCC827 and A549, were obtained from the BeNa Culture Collection (Beijing, China). BEAS-2B and A549 cells were maintained in Dulbecco’s modified Eagle’s medium (DMEM)/F12 containing 10% heat-inactivated fetal bovine serum, 100 IU/ml penicillin, and 10 g/ml streptomycin. The H125 and HCC827 cell lines were both incubated in RPMI1640 medium containing 10% heat-inactivated fetal bovine serum, 100 IU/ml penicillin, and 10 g/ml streptomycin. All cell lines were incubated in a 95% air and 5% carbon dioxide (CO_2_) atmosphere at 37 °C.

### Western blot analysis

Approximately 1 × 10^7^ cells were solubilized in lysis buffer purchased from the Beyotime Institute of Biotechnology (Shanghai, China). Twelve percent SDS-PAGE was utilized to separate the proteins. Afterwards, approximately 60 μg of protein was transferred to a polyvinylidene difluoride (PVDF) membrane. Then, the membrane adsorbing the proteins was incubated with TBST buffer (Tween 20) at room temperature containing 5% nonfat milk. After 3 h, the membrane was incubated with primary antibodies for 3 h at room temperature. After washing with TBST buffer (Tween 20), the membranes were treated with a matched secondary antibody for 1 h. The following primary antibodies were used: rabbit anti-SMAD3 (1:5000 dilution, ab40854) and rabbit anti-PAX6 (1:1000 dilution, ab5790); the secondary antibody was goat anti-rabbit labeled with HRP (horseradish peroxidase) (1:5000 dilution, ab205718). All antibodies were obtained from Abcam (Cambridge, MA, USA). An ECL kit and the Image-Pro plus software, version 6.0, from Media Cybernetics (Rockville, MD, USA) were used to determine the chemiluminescent and relative protein expression, respectively, which was represented as the density ratio vs. GAPDH.

### Cell transfection

To knock down *SMAD3 and PAX6*, Lipofectamine 2000 (Invitrogen, Life Technologies, Carlsbad, CA, USA) was used to transfect A549 and H125 cells with siRNA-SMAD3. Two different *SMAD3-*specific siRNAs (GenePharma, Shanghai, China), si-SMAD3 #1 and si-SMAD3 #2, and a PAX6 *siRNA*, si-PAX6 (GenePharma, Shanghai, China), were transfected into the cells to knockdown gene expression. Si-NC, a scrambled siRNA, was used as a control. The pcDNA3.1-PAX6 (p-PAX6) and pcDNA3.1 (GenePharma, Shanghai, China) control vectors were also transfected with Lipofectamine 2000 according to the manufacturers’ instructions. The siRNA sequences are shown in Additional file 1: Table S1.

### Double luciferase reporter assay

The *PAX6* promoter region was cloned into the PGL3-luc luciferase reporter vector to construct the PAX6*-*luc luciferase reporter vector. To investigate the relationship between *SMAD3* and *PAX6*, pCMV-SMAD3 was cotransfected with the PAX6*-*luc reporter plasmid and pRL-TK plasmid as an internal control using Lipofectamine™ 2000. Luciferase activities were detected 48 h after transfection with the Dual-luciferase Reporter Assay System (Promega, WI, USA). Firefly luciferase activity was normalized to Renilla luciferase activity. The primers for plasmid construction are shown in Additional file 2: Table S2.

### Real-time RT-PCR

The RNeasy® Mini Kit (Qiagen®, Venlo, Netherlands) was performed to extract total RNA from collected tissues or cultured cells, which was then reverse transcribed into cDNA using the M-MLV reverse transcriptase (Invitrogen). SYBR Premix Ex Taq from TaKaRa Biotechnology (Tokyo, Japan) was used to quantify *SMAD3* and *PAX6* expression*. GUSB* and *GAPDH* were both used as internal controls for the tissues and cells, respectively. The primer probes were purchased from GenePharma (Shanghai, China). All data were quantified with the 2^−ΔΔCT^ method. The qPCR primers are shown in Additional file 2: Table S2.

### ChIP assay

One percent formaldehyde was used to treat and crosslink cells from each group for approximately 10 min at room temperature. After lysis, the cells were sonicated to breakdown the chromatin into 200 bp to 1 kb fragments. Antibodies specific to SMAD3 (ab28379) or IgG (ab172730), as a negative control (Abcam, Cambridge, MA, USA), were used to immunoprecipitate the chromatin by generating antigen-antibody complexes. Afterwards, the complexes were collected by protein A agarose beads (Merck Millipore, Billerica, MA, USA), followed by washing to remove any nonspecific binding. The DNA was eluted from the immunoprecipitated complexes on the agarose beads with 0.1 M NaHCO_3_ and 1% SDS. The primers for ChIP-qPCR are shown in Additional file 2: Table S2.

### CCK-8 assay

The Cell Counting Kit-8 (CCK-8) assay was used to evaluate cell viability and proliferation. Briefly, the cell lines were seeded onto 96-well plates (3000 cells/well, Corning, NY, USA) and incubated for the indicated time points (0, 24, 48, 72, or 96 h). Next, 10 μL of CCK-8 solution was added to each well and the cells were incubated in the dark at 37 °C for 2 h. Afterwards, the absorbance was detected at 490 nm to assess cell viability.

### Transwell migration assay

In total, 1 × 10^5^ cells in 250 μL of medium containing 0.1% FBS were seeded into 24-well-plates (Corning, NY, USA) with noncoated inserts for the migration assay. Then, 750 μL medium supplemented with 10% FBS was added into the lower chamber. After incubating the cells for 24 h, nonmigrating cells in the upper chamber were washed away and the cells in the lower chamber were fixed with cold methanol. Hoechst 33258 and a Zeiss Axiophot epifluorescence microscope purchased from QImaging (Surrey, BC) were used to stain and count cells in 5 random visible fields, respectively.

### Transwell invasion assay

Serum-free medium was added to dilute the Matrigel (1:7), and then 50 μL of diluted Matrigel was inoculated into each chamber. The prepared chambers were placed in an incubator at 37 °C for 4 h for the following experiments. In total, 1 × 10^5^ cells in 250 μL of medium containing 0.1% FBS were seeded into the apical chamber covered by diluted Matrigel, while 500 μL of culture medium with 10% FBS was added to the basolateral chamber. After incubating for 36 h, the nonmigrating cells in the upper chamber were washed away and the cells in the lower chamber were fixed with cold methanol. Hoechst 33258 and a Zeiss Axiophot epifluorescence microscope purchased from QImaging (Surrey, BC) were used to stain and count cells in 5 random visible fields, respectively.

### Colony forming assay

After trypsinization, single-cell suspensions were collected followed by seeding of approximately 300 cells/well into 6-well-plates (Corning, NY, USA). All plates were cultured to form visible colonies at 37 °C. Afterwards, the cells in the plates were fixed with methanol and counted using 0.5% crystal violet.

### Statistical analysis

All measurements were performed in triplicate. The Student’s t-test was employed to analyze the differences between two groups with *P* < 0.05 considered to be significant. The differences among the groups of samples were accomplished by one-way ANOVA. Data are presented as the mean ± SD.

## Results

### The high expression of *SMAD3 and PAX6* in NSCLC tissues and cells

**Fig. 1:**
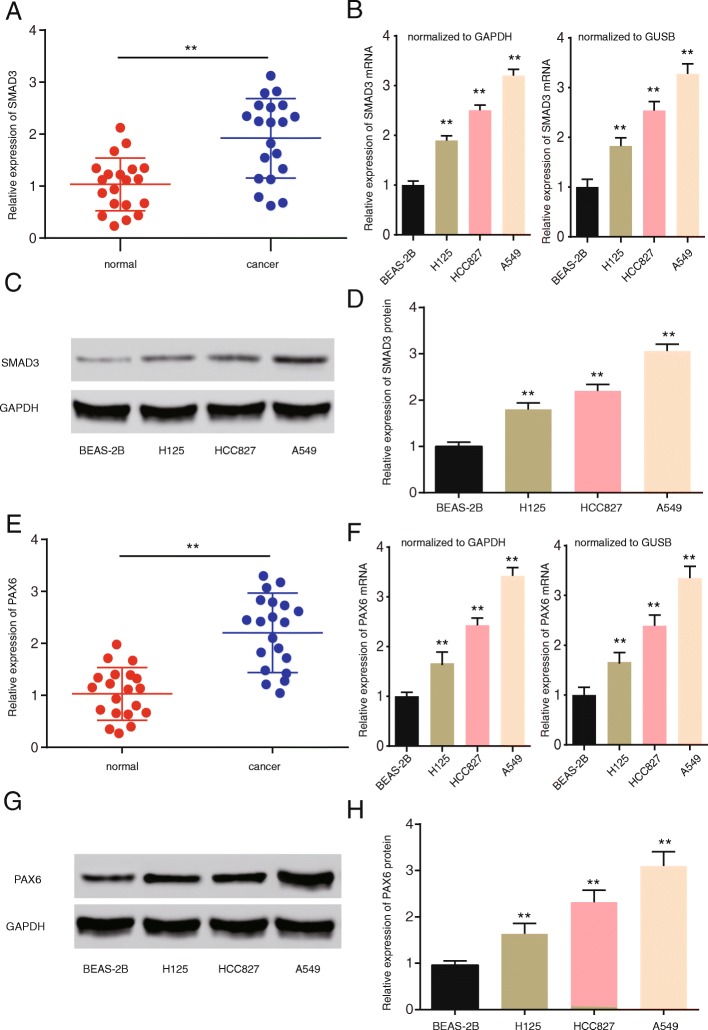
*SMAD3 and PAX6* are highly expressed in NSCLC tissues and cells. **a** *SMAD3* expression levels in NSCLC tissues compared with the normal groups (*n* = 20). **b** *SMAD3* mRNA levels in the BEAS-2B, H125, HCC827 and A549 cell lines normalized to GAPDH (left panel) and GUSB (right panel) (*n* = 3). (**c** and **d**) *SMAD3* protein levels in the BEAS-2B, H125, HCC827 and A549 cell lines (*n* = 3). **e** *PAX6* expression levels in the NSCLC tissues compared with the normal groups (*n* = 20). **f** *PAX6* mRNA levels in the BEAS-2B, H125, HCC827 and A549 cell lines normalized to GAPDH (left panel) and GUSB (right panel)(*n* = 3). **g** and **h** *PAX6* protein levels in the BEAS-2B, H125, HCC827 and A549 cell lines (*n* = 3). Data represent the mean ± SD. **P < 0.01 compared with the control group

QRT-PCR was utilized to investigate *SMAD3* and PAX6 expression levels in 20 normal and 20 NSCLC cancer tissues. Figure 1a and e suggest that *SMAD3* and *PAX6* are up-regulated in NSCLC tissues compared with normal tissues (*P* < 0.01). Similar *SMAD3* and *PAX6* mRNA and protein expression was observed in the four cell lines, including the normal BEAS-2B human lung epithelial cells and H125, HCC827 and A549 cancer cell lines. Figure 1b and f demonstrated that *SMAD3* and *PAX6* mRNA levels were upregulated in cancer cell lines compared with the normal cell line (*P* < 0.01). Furthermore, higher *SMAD3* and *PAX6* protein expression was observed in the cancer cell lines compared with the healthy human lung epithelial cells (Fig. 1c, d, g, h, Additional file 3: Figure S1a and b) (*P* < 0.01). Thus, NSCLC tissues and cells displayed high *SMAD3* and *PAX6* expression compared with control tissues and cells.

### Knocking down *SMAD3* lowered *PAX6* expression

**Fig. 2:**
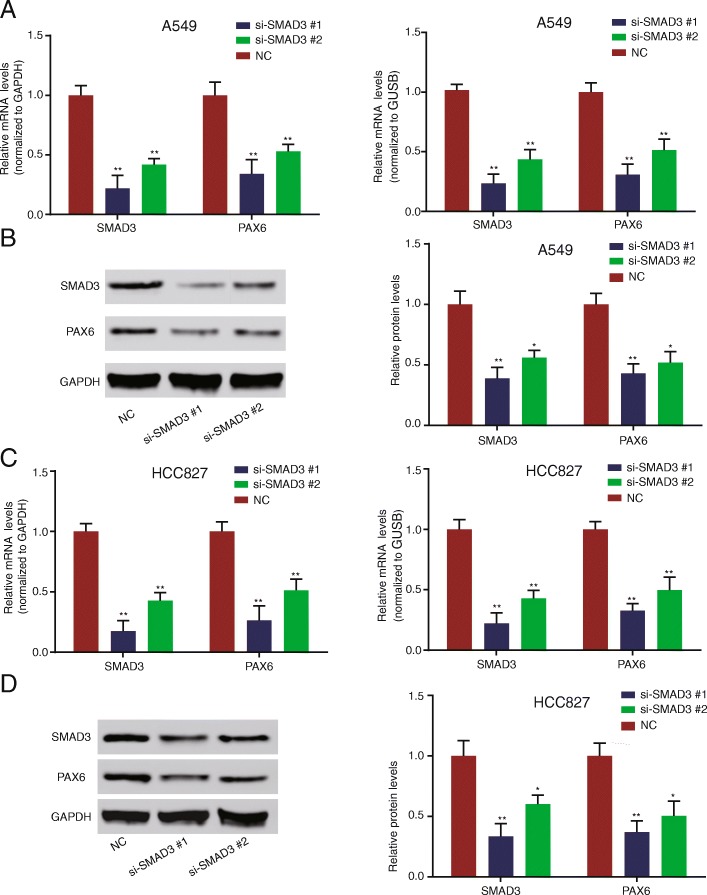
*SMAD3* knockdown decreased *SMAD3* and *PAX6* expression levels. **a** *SMAD3* and *PAX6* mRNA expression levels in A549 cells transfected with siRNA and normalized to GAPDH (left panel) and GUSB (right panel). **b** SMAD3 and PAX6 protein expression levels in A549 cells transfected with siRNA (*n* = 3). **c** *SMAD3* and *PAX6* mRNA expression levels in HCC827 cells transfected with siRNA normalized to GAPDH (left panel) and GUSB (right panel). **d** SMAD3 and PAX6 protein expression levels in HCC827 cells transfected with siRNA. Data represent the mean ± SD. *P < 0.05 and **P < 0.01 compared with the control group

To investigate the effect of SMAD3 on *PAX6* expression, *SMAD3* was knocked down by siRNA transfection, followed by the detection of *PAX6* and *SMAD3* expression levels by qRT-PCR and Western blot in the A549 and HCC827 cell lines. The results demonstrated that silencing *SMAD3* downregulated *PAX6*, as shown by the lower *PAX6* and *SMAD3* mRNAs and proteins in the si-*SMAD3*#1 and si-SMAD3#1 groups compared with the NC group in the A549 (Fig. 2a, b and Additional file 3: Figure S1c, all *P* < 0.05) and HCC827 (Fig. 2c, d and Additional file 3: Figure S1d) cell lines.

### *The SMAD3* protein regulated *PAX6* transcription via binding its promoter

**Fig. 3:**
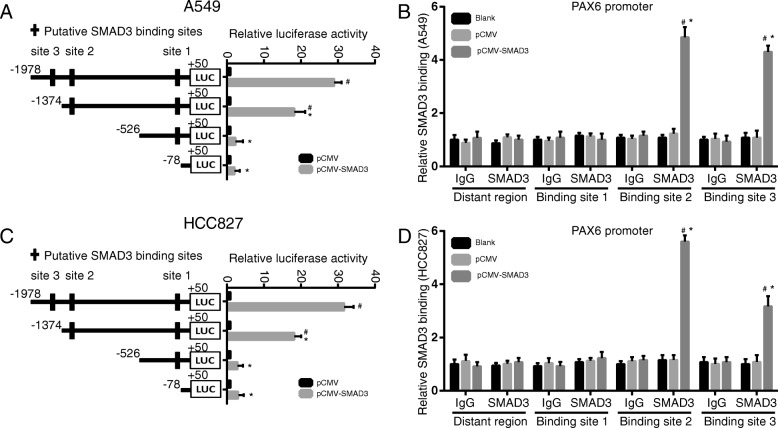
*SMAD3 knockdown* inhibited *PAX6* transcription in A549 and HCC827 cells. **a** Deletion mutation analyses identified the FoxC1-responsive regions in the CCL2 promoter in A549 cells. Serially truncated PAX6 promoter constructs were cotransfected with pCMV-SMAD3 and relative luciferase activities were determined. The schematic constructs are shown (left) and the bar graphs represent the relative levels of luciferase activity in each of the samples (right). #*P* < 0.05 compared with the pCMV group. *P < 0.05 compared with the group with the − 1978 ~ + 50 regions cloned into the luciferase reports plasmids. **b** ChIP assay demonstrated the direct binding of SMAD3 to the PAX6 promoter in A549 cells. Real-time PCR was performed to detect the amounts of immunoprecipitated products. The cells were crosslinked and the chromatin was immunoprecipitated using anti-SMAD3 or control antibodies. #*P* < 0.05 compared with the pCMV group. *P < 0.05 compared with the distant region. **c** Deletion mutation analyses identified FoxC1-responsive regions in the CCL2 promoter in HCC827 cells. #*P* < 0.05 compared with the pCMV group. *P < 0.05 compared with the group with the − 1978 ~ + 50 regions cloned into the luciferase reports plasmids. **d** ChIP assay demonstrated the direct binding of SMAD3 to the PAX6 promoter in HCC827 cells. #*P* < 0.05 compared with the pCMV group. *P < 0.05 compared with the distant region

Dual luciferase assay and ChIP assay were exploited to assess the effects of *SMAD3* on *PAX6* promoter activity. Sequence analysis revealed 3 putative SMAD3 binding sites in the PAX6 promoter. Serial deletion showed that the second and third SMAD3 binding sites were critical for SMAD3-induced PAX6 trans-activation in A549 and HCC827 cells (Fig. 3a and c). ChIP assay further confirmed that SMAD3 directly binds to the PAX6 promoter in A549 and HCC827 cells (Fig. 3b and d). These studies demonstrated that PAX6 was a direct transcriptional target of SMAD3 in both cell lines.

### SMAD3 downregulation on the physiological abilities of A549 and HCC827 cells

**Fig. 4:**
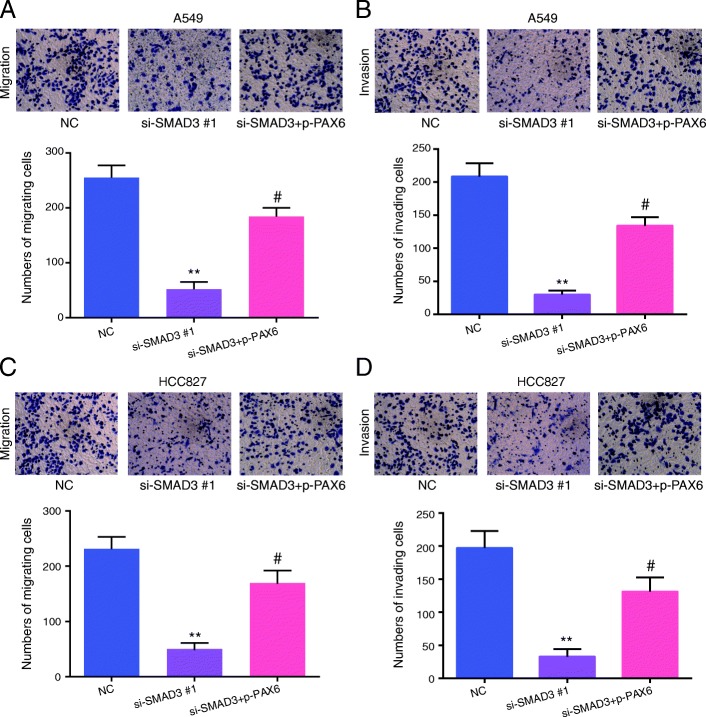
*SMAD3* downregulation decreased the cell migration and invasion of A549 and HCC827 cells. **a** SMAD3 downregulation inhibited the migration of A549 cells. **b** SMAD3 downregulation inhibited the invasion capacity of A549 cells. **c** SMAD3 downregulation inhibited the migration of HCC827 cells. **d** SMAD3 downregulation inhibited the invasion capacity of HCC827 cells. All experiments were performed in triplicate. Data represent the mean ± SD. NC group: cells were transfected with scrambled siRNA. **P < 0.01 compared with the NC group. #*P* < 0.05 compared with the si-SMAD3 #1 group

**Fig. 5:**
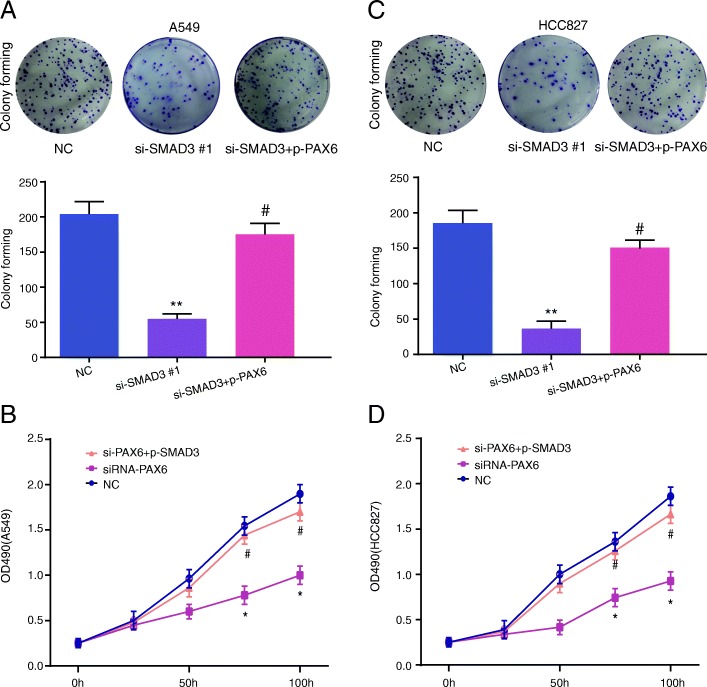
*SMAD3* downregulation decreased the colony forming capacity and cell viability of A549 and HCC827 cells. **a** The colony forming capacity of A549 cells was detected by colony forming assay. **b** The cell viability of A549 cells was determined by CCK-8 assay. **c** The colony forming capacity of HCC827 cells was detected by colony forming assay. **d** The cell viability of HCC827 cells was determined by CCK-8 assay. All experiments were performed in triplicate. Data represent the mean ± SD. *P < 0.05 and **P < 0.01 compared with the NC group. ^#^*P* < 0.05 compared with the si-SMAD3 group

To evaluate A549 and HCC827 cell proliferation, invasion and migration after *SMAD3* silencing and explore whether SMAD3 exerts its biological activity through PAX6, we performed CCK-8, colony forming and Transwell assays. Figure 4a and b demonstrated that the A549 cell line si-SMAD3#1 group showed lower cell migration (Fig. 4a) and invasion (Fig. 4b) compared with the control group (*P* < 0.01). Moreover, when cells in the si-SMAD3#1 group were cotransfected with pc-DNA3.1-PAX6 (si-SMAD3 + p-PAX6), cell migration and invasion were up-regulated compared with the si-SMAD3#1 group (*P* < 0.01), which suggests that PAX6 overexpression could reverse the cellular physiological abilities inhibited by SMAD3 knockdown. Similar results were observed in the HCC827 cell line, which are shown in Fig. 4c and d. Similarly, the si-SMAD3#1 group cells showed lower colony formation abilities compared with control group in both the A549 and HCC827 cell lines (Figs. 5a and b), and upregulating *PAX6* expression improved this situation (*P* < 0.01). The CCK-8 assay showed that downregulation of *SMAD3* suppressed cell viability compared with control group and that enhanced *PAX6* expression improved the inhibition of si-SMAD3 on cell viability in A549 and HCC827 cells (Figs. 5c and d, *P* < 0.05). These results demonstrated that the downregulation of SMAD3 acted as an inhibitor of A549 and HCC827 cell physiological abilities, which could be partially reversed by PAX6 overexpression.

### SMAD3 and PAX6 knockdown to explore their relevance in the A549 and HCC827 cell lines

**Fig. 6:**
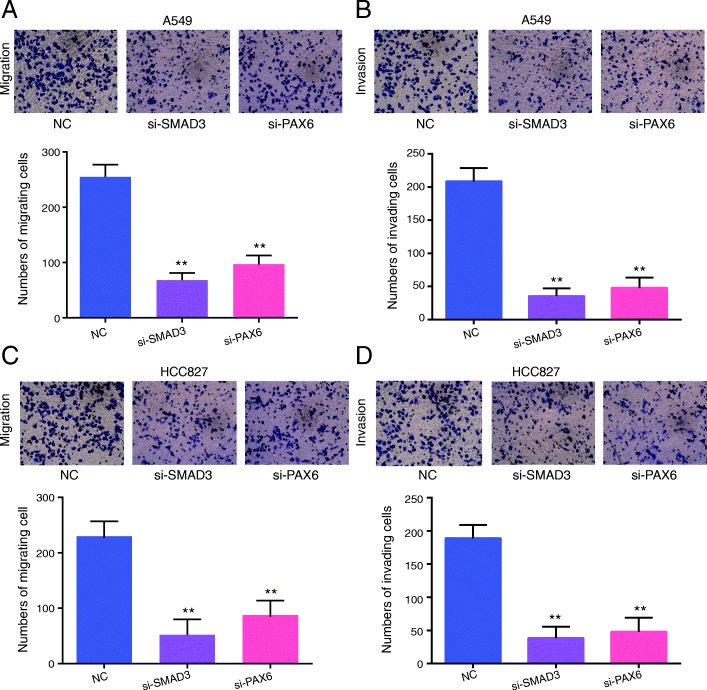
*PAX6* downregulation decreased the cell migration and invasion of A549 and HCC827 cells. **a** PAX6 downregulation inhibited the migration of A549 cells. **b** PAX6 downregulation inhibited the invasion capacity of A549 cells. **c** PAX6 downregulation inhibited the migration of HCC827 cells. **d** PAX6 downregulation inhibited the invasion capacity of HCC827 cells. All experiments were performed in triplicate. Data represent the mean ± SD. **P < 0.01 compared with the NC group

**Fig. 7:**
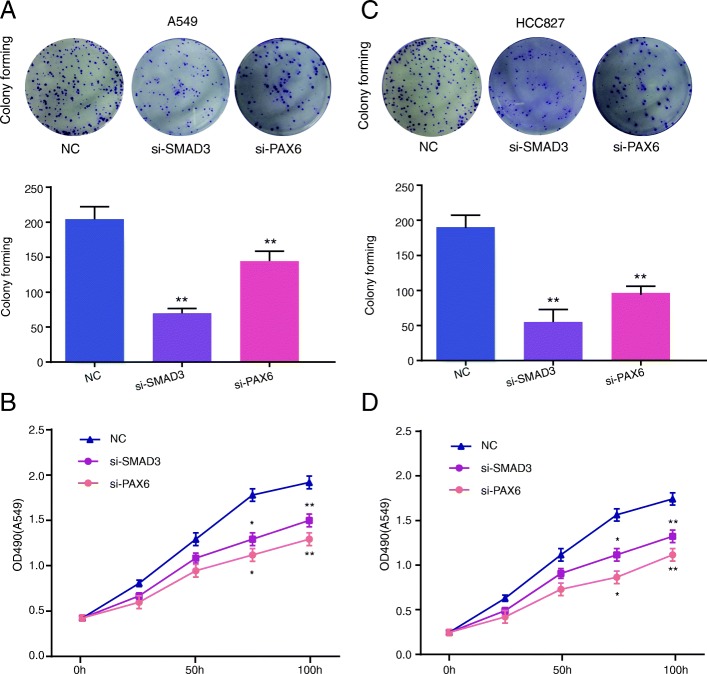
*PAX6* downregulation decreased the colony forming capacity and cell viability of A549 and HCC827 cells. **a** The colony forming capacity of A549 cells after transfection with si-SMAD3 or si-PAX6 was detected by colony forming assay. **b** The cell viability of A549 cells after transfection with si-SMAD3 or si-PAX6 was determined by CCK-8 assay. **c** The colony forming capacity in HCC827 cells after transfection with si-SMAD3 or si-PAX6 was detected by colony forming assay. **d** The cell viability of HCC827 cells after transfection with si-SMAD3 or si-PAX6 was determined by CCK-8 assay. All experiments were performed in triplicate. Data represent the mean ± SD. *P < 0.05 and **P < 0.01 compared with the NC group

To further explore the relevance and functional effects of PAX6, we depleted PAX6 and SMAD3 through the transfection of si-SMAD3 and si-PAX6, respectively (Figs. 6 and 7). We demonstrated that A549 cell migration (Fig. 6a), invasion (Fig. 6b), colony forming ability (Fig. 7a) and cell viability (Fig. 7b) were repressed in the si-PAX6 group, which show lower decreasing trends compared with the si-SMAD3 group, though there were no significant differences. Similar experiments were also performed in the HCC827 cell line (Figs. 6c and d and 7c and d). *PAX6* showed similar functions as *SMAD3*, but the tendency might be weaker. Thus, *SMAD3* might partially target *PAX6* to regulate cell migration, invasion, proliferation and viability in NSCLC patients with positive correlations.

## Discussion

In the present study, *SMAD3* expression levels were evaluated in healthy and NSCLC tissues and cells, showing its high expression. This high *SMAD3* expression might play a crucial role in the development of NSCLC through the targeted modulation of *PAX6* expression, resulting in the enhancement of cell migration, invasion, proliferation and viability.

In a previous study, *SMAD3* was significantly associated with human osteoarthritis and upregulated in human osteoarthritic cartilage, though not due to DNA methylation in the promoter region [20, 21]. Qian et al. showed that enhancing *SMAD3* phosphorylation was associated with high metastatic potential in nonsmall cell lung cancer by downregulating E-cadherin [22]. Furthermore, Yang et al. demonstrated that inhibiting SMAD-dependent signaling in NSCLC might repress the epithelial-mesenchymal transition and cell invasion [23]. However, *SMAD3* is also likely to play a role as a cancer suppressor in NSCLC through other mechanisms. For example, in the study by Samanta et al., smoking promotes tumorigenicity and results from the reduction in *SMAD3* expression along with the abrogation of TGF-β-mediated growth inhibition [9]. It was also shown that NORAD (also known as LINC00657 or LOC647979), a cytoplasmic long noncoding RNA, indirectly interacts between importin β1 and SMAD3 in NSCLC, and is widely considered as a regulator of TGF-β signaling [10]. Therefore, the function of *SMAD3* in NSCLC might have a dual character, which deserves deeper investigation. In our study, high *SMAD3* expression was found in NSCLC cells and tissues and acts as an oncogene.

Understanding on the effects of deregulated *PAX6* expression on the development of NSCLC remains insufficient, and few studies have focused on the relationship between *SMAD3* and *PAX6*. In accordance with the study by Zhao et al., *PAX6* expression was significantly enhanced in NSCLC tissues compared with matched adjacent tissues and was associated with promoting cell cycle progression [24]. Moreover, Zhang et al. demonstrated that *PAX6* gene methylation in NSCLC is usually associated with poor prognosis in NSCLC via a methylation-specific PCR assay [25]. Therefore, there is research that strongly supports the assumption that PAX6 is a valid and positive prognostic marker in node-positive NSCLC patients.

The relevance between SMAD3 and PAX6 has been explored. It was shown that SMAD3 interacted with PAX6 and repressed autoregulation of the PAX6 P1 promoter in NSCLC cells. Therefore, in the present study, *SMAD3* and *PAX6* and their interactions were deeply investigated; we found that *SMAD3* expression positively promoted *PAX6* transcription, which then regulated NSCLC cell migration, proliferation and viability. However, as the transfection efficiency and quantitative examination were different, comparing the effects between knocking down SMAD3 and PAX6 might make little sense, although we did find that si-SMAD3 was more effective at affecting cell migration, invasion, colony formation and proliferation than si-PAX6.

Generally, NSCLC tissues and cell lines had higher *SMAD3* and *PAX6* expression level than normal tissues and cell lines. Moreover, *SMAD3* downregulation could inhibit *PAX6* transcription, resulting in the suppression of *PAX6* expression and hindering cell migration, invasion, proliferation and viability in NSCLC cells. Based on these findings, inhibiting *SMAD3* and *PAX6* should be further explored and may become a promising mechanism for treating nonsmall cell lung cancer in the future.

## Conclusions

SMAD3 and PAX6 were upregulated in lung cancer tissues and cancer cells. Knocking down *SMAD3* and *PAX6* by transfection with specific siRNAs suppressed the expression of *SMAD3* and *PAX6* mRNA and protein levels. SMAD3 could promote *PAX6* transcriptional activity via binding to its promoter. Reduced expression of SMAD3 downregulated PAX6 at the mRNA and protein levels while also decreasing cell migration, invasion, proliferation and viability in NSCLC cells. PAX6 overexpression altered the inhibitory effects of si-SMAD3 on cell migration, invasion, proliferation and viability. PAX6 knockdown alone could also inhibit *A549* and HCC827 cell functions*.* Thus*, SMAD3* promotes the progression of nonsmall cell lung cancer by upregulating *PAX6* expression.

## Additional files

Additional file 1: **Table S1.** siRNAs sequences. (DOCX 15 kb)

Additional file 2: **Table S2.** Primer sequences used in the study. (DOCX 18 kb)

Additional file 3: **Figure S1.** Uncropped blot of the result of western blot. (A) the uncropped blot of SMAD3 protein in Fig 1C. (B) the uncropped blot of PAX6 protein in Fig 1G. (C) the uncropped blot of SMAD3 and PAX6 protein in Fig. 2B. (D) the uncropped blot of SMAD3 and PAX6 protein in Fig 2D.(TIF 6168 kb)
