# Supplementary material for: Investigating the mechanism by which SMAD3 induces PAX6 transcription to promote the development of non-small cell lung cancer
Source: Respir Res. 2018 Dec 29;19:262. doi: 10.1186/s12931-018-0948-z (PMC6311080; doi:10.1186/s12931-018-0948-z)
Supplement: Supplementary file 1 — Table S1. siRNAs sequences. (DOCX 15 kb) [file 12931_2018_948_MOESM1_ESM.docx]

**Table S1 siRNAs sequences**

| Gene | Strand | Sequence |
| --- | --- | --- |
| si-SMAD3 #1 | Sense | 5′‐CGUCAACACCAAGUGCAUCTT‐3′ |
|  | Antisense | 5′‐GAUGCACUUGGUGUUGACGTT‐3′ |
| si-SMAD3 #2 | Sense | 5′‐GCAACCUGAAGAUCUUCAATT‐3′ |
|  | Antisense | 5′‐UUGAAGAUCUUCAGGUUGCTT‐3′ |
| si-PAX6 | Sense | 5′‐CGUGUCCAACGGAUGUGUGAGUAAA‐3′ |
|  | Antisense | 5′‐GAAAGAGUUUGAGAGAACCCAUUAU‐3′ |
| si-NC | Sense | 5′‐UUCUCCGAACGUGUCACGUTT‐3′ |
|  | Antisense | 5′‐ACGUGACACGUUCGGAGAATT‐3′ |
