# Supplementary material for: Investigating the mechanism by which SMAD3 induces PAX6 transcription to promote the development of non-small cell lung cancer
Source: Respir Res. 2018 Dec 29;19:262. doi: 10.1186/s12931-018-0948-z (PMC6311080; doi:10.1186/s12931-018-0948-z)
Supplement: Supplementary file 2 — Table S2. Primer sequences used in the study. (DOCX 18 kb) [file 12931_2018_948_MOESM2_ESM.docx]

**Table S2 Primer sequences used in the study.**

| Primer name | Primer sequences (5’-3’) |
| --- | --- |
| Primers for RT-PCR |  |
| SMAD3 sense | GGCTGGAAGAAGGGCGAGCAG |
| SMAD3 antisense | CAGGGACCTGGGGATGGTGATGC |
| PAX6 sense | CACACCGGTTTCCTCCTTCA |
| PAX6 antisense | GGCAGAGCGCTGTAGGTGTT |
| GUSB sense | CCCACTCAGTAGCCAAGTCA |
| GUSB antisense | CACGCAGGTGGTATCAGTCT |
| GAPDH sense | TGCACCACCAACTGCTTAGC |
| GAPDH antisense | GGCATGGACTGTGGTCATGAG |
| Primers for PAX6 promoter construct: |  |
| (-1978/+50) PAX6 sense | TATAGAGCTCGGGATTTGCGCACACTTAATG |
| (-1374/+50) PAX6 sense | TATAGAGCTCAGTCCAGCTAGGAGCTGTCCG |
| (-526/+50) PAX6 sense | TATAGAGCTCCCCAGCTAGTGACTTGCGGGC |
| (-78/+50) PAX6 sense | TATAGAGCTCCATGACGTCACGCGGGCC |
| antisense | TATAACGCGTACGCGAGGACCTGCCC |
| Primers used for ChIP in the PAX6 promoter: |  |
| SMAD3 binding site 1 sense | AGTCGGCGCAGAGCTGTGCCC |
| SMAD3 binding site 1 antisense | CCAGCGCCGTCCTCATTGGCTG |
| SMAD3 binding site 2 sense | GGATCAAGGCGCGCAGGGAC |
| SMAD3 binding site 2 antisense | CCTTTAGGCAGCTCTCGTGGAGACCC |
| SMAD3 binding site 3 sense | CAGCCGAGCAGGGCGACAGGA |
| SMAD3 binding site 3 antisense | TGTTAAGGCCTAGGAGCGCCC |
