# Supplementary figures and images for: Investigating the mechanism by which SMAD3 induces PAX6 transcription to promote the development of non-small cell lung cancer
Source: Respir Res. 2018 Dec 29;19:262. doi: 10.1186/s12931-018-0948-z (PMC6311080; doi:10.1186/s12931-018-0948-z)

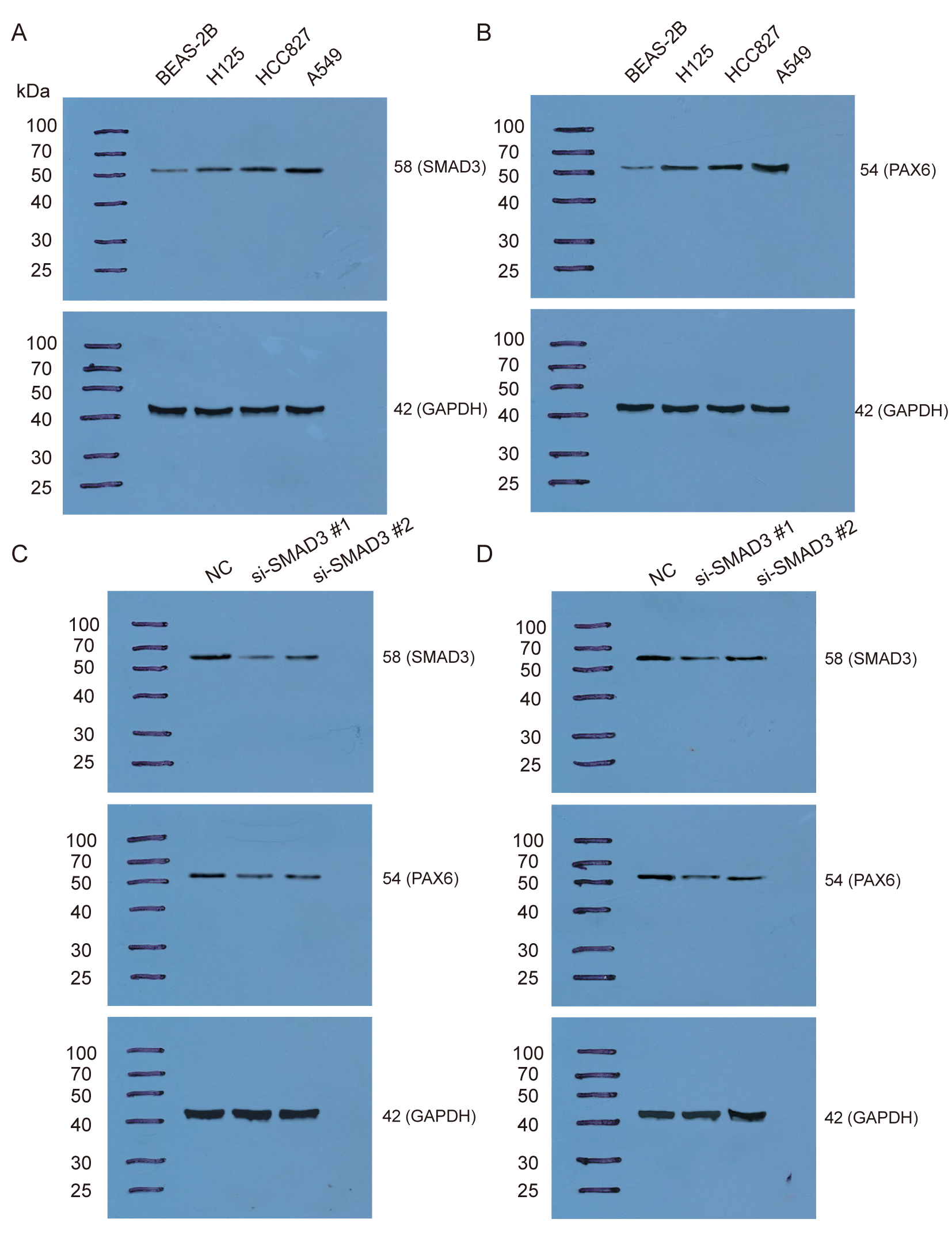

Supplement: Supplementary file 3 — Figure S1. Uncropped blot of the result of western blot. (A) the uncropped blot of SMAD3 protein in Fig 1C. (B) the uncropped blot of PAX6 protein in Fig 1G. (C) the uncropped blot of SMAD3 and PAX6 protein in Fig. 2B. (D) the uncropped blot of SMAD3 and PAX6 protein in Fig 2D.(TIF 6168 kb) [file 12931_2018_948_MOESM3_ESM.tif]
